# Editorial: Loneliness among youth and young adults

**DOI:** 10.3389/fpsyt.2026.1879683

**Published:** 2026-06-09

**Authors:** Jessica Hemberg, Renae Schmidt, Viviana Horigian

**Affiliations:** 1 Abo Akademi, Turku, Finland; 2 Department of Health Sciences, Faculty of Science and Engineering, Abo Akademi University, Turku, Finland; 3 Department of Public Health Sciences, Leonard M. Miller School of Medicine, University of Miami, Miami, FL, United States

**Keywords:** adolescents, loneliness, mental health, social connectedness, social media, young adults

Recent research indicates a rise in loneliness and diminished social connectedness among adolescents and young adults. This trend has been linked to adverse health outcomes, including mental health issues and higher mortality rates. The body of work reviewed in this Research Topic demonstrates loneliness as a foundational mechanism through which social, psychological, and behavioral forces converge, shaping young people’s mental health, academic trajectories, and risk behaviors. Loneliness, this work collectively argues, is a central organizing force in the mental health landscape of young people today. Understanding what loneliness is doing in young people’s lives, and how to address it, requires drawing on the full breadth of perspectives the field has to offer.

Schmidt et al., in their qualitative study of young adult women using anonymous asynchronous online focus groups, highlight rising anxiety, depression, loneliness, and social media addiction in this population. Their study reveals a landscape marked by stigma, unmet emotional needs, and a search for safe spaces to be heard. Participants described both the harms and possibilities of digital environments – spaces that can amplify comparison and exclusion, but also offer anonymity, validation, and connection when other types of support fall short. This duality is echoed across multiple studies and complicates any simplistic narrative that positions technology as either wholly harmful or beneficial.

Indeed, the relationship between digital life and loneliness is consistently mediated by deeper psychological processes. Fang et al. demonstrate that problematic mobile social network use contributes to social anxiety through increased loneliness and reduced self-concept clarity. Similarly, Aslan and Polat show that social media addiction undermines academic self-efficacy and success, with loneliness, depression, and life dissatisfaction acting as key intermediaries. These findings suggest that it is not mere usage, but the erosion of meaningful connection and stable identity that drives negative outcomes.

Beyond the digital sphere, loneliness also mediates the effects of how young people see themselves and their place in the world. Hu et al. provide compelling longitudinal evidence that body weight misperception is linked to poorer academic performance, with loneliness acting as a crucial pathway. Misperceiving one’s body is not just an internal cognitive distortion – it reshapes social participation, peer relationships, and ultimately academic engagement. In a different domain, Liang et al. show that participation in competitive sports can reduce self-harm among adolescents, but only insofar as it enhances public belonging and reduces loneliness. These findings converge on a central insight: belonging is not a byproduct of participation; it is the mechanism through which participation becomes protective.

Clinical and at-risk populations highlight the stakes more starkly. Dargél et al., in their scoping review, identify loneliness as both highly prevalent and particularly damaging among adolescents and young adults with bipolar disorder. Loneliness in this group is not episodic but chronic, intensifying mood instability and complicating recovery. Similarly, Padmanabhanunni and Pretorius demonstrate that loneliness is closely tied to internalizing disorders, with ego-resilience playing a dual role as both mediator and moderator. Individuals with higher resilience are better equipped to buffer the psychological impact of loneliness, pointing to the importance of adaptive coping capacities alongside social interventions.

At the population level, the scope of the issue becomes even clearer. Verity et al. synthesize evidence showing that loneliness peaks during key developmental transitions and is shaped by intersecting contexts – family dynamics, peer relationships, and school environments. Loneliness is not randomly distributed; it follows predictable patterns tied to structural and developmental pressures. Burke et al. deepen this perspective by examining how children and adolescents themselves describe coping with loneliness. While many demonstrate insight – seeking connection, engaging in distraction, or reframing their thoughts – their strategies are often constrained by the very environments that produce loneliness in the first place. In other words, young people are lacking opportunity for connection.

Encouragingly, emerging intervention research suggests that loneliness is modifiable, though not easily so. Ma et al. show that addressing loneliness within primary care settings for emerging adults is both feasible and acceptable, pointing to healthcare systems as underutilized sites for social and psychological intervention. Wang et al. further suggest that cultivating a growth mindset can improve well-being in social interactions, offering a cognitive pathway to counteract loneliness by reshaping how individuals interpret and respond to social challenges. These approaches, while promising, remain small in scale and underscore the need for broader, systemic implementation.

The methodological strengths demonstrated throughout these articles are significant. Qualitative approaches—including arts-based and anonymous asynchronous online focus groups—successfully surface youth voices that are often muted in survey-based research. These methods appear particularly well-suited for studying loneliness, stigma, and gendered experiences, allowing participants to articulate nuance around social disconnection, trust, and help-seeking preferences. Quantitative studies complement this by identifying mediating and chain-mediating pathways reinforcing the value of integrating psychological processes into loneliness models.

At the same time, there are shared methodological challenges that cut across studies. Most rely on cross-sectional designs, limiting causal inference and obscuring how loneliness unfolds over time or in response to interventions. Heavy reliance on self-reported data raises questions about social desirability or accuracy and risks of labeling some normal behaviors as pathological. Samples are often context-specific constraining generalizability while underscoring the importance of contextualized interpretation rather than universal claims.

Future directions point toward several priorities in research. Longitudinal and mixed-methods designs are needed to trace developmental trajectories and identify sensitive periods for intervention. Research should test multi-level interventions that simultaneously target individual skills, interpersonal environments, and social infrastructure. Improved measurement, including contextualized loneliness assessments and objective or ecological indicators of social interaction—will be critical. Finally, advancing equity requires intersectional approaches that examine how loneliness is shaped by gender, race, sexual identity, and social position, and how digital spaces might be redesigned to support—rather than erode—meaningful connection.

Addressing youth mental health requires directly tackling loneliness. Interventions should focus on building environments of belonging across schools, healthcare, communities, and digital platforms. This means fostering authentic interactions online, integrating social connection into routine care, and creating spaces that welcome diverse identities.

